# Quality of life for kidney transplant recipients and hemodialysis patients in Palestine: a cross-sectional study

**DOI:** 10.1186/s12882-021-02412-z

**Published:** 2021-06-03

**Authors:** Adnan Lutfi Sarhan, Raya H. Jarareh, Mujahed Shraim

**Affiliations:** 1 Department of Public Health, Faculty of Medicine and Health Sciences, An-Najah National University, Ramallah, Palestine; 2 Palestine Medical Complex, Ministry of Health, Ramallah, Palestine; 3 Department of Public Health, College of Health Sciences, QU Health, Qatar University, PO Box. 2713, Doha, Qatar

**Keywords:** Quality of life, Renal Dialysis, Kidney transplantation, Cross-sectional studies

## Abstract

**Background:**

Health related quality of life (HRQOL) is an important indicator of medical treatment and is a strong predictor of disability and mortality. The literature has shown mixed evidence about whether kidney transplantation improves HRQOL compared with other renal replacement modalities. The aim of this study was to compare the HRQOL in kidney transplant recipients (KTRs) and hemodialysis (HD) patients.

**Methods:**

A cross-sectional study of 100 KTRs and 272 HD patients from two central kidney units in the West Bank, Palestine. The HRQOL was assessed using the Short Form-36 Health Survey. Multivariable linear regression was used to estimate differences in mean HRQOL scores between KTRs and HD patients.

**Results:**

As compared to HD patients, KTRs had higher clinically important HRQOL in main domains and subscales of the SF-36 including physical functioning, role-physical, bodily pain, general health, vitality, social functioning, role-emotional, mental health, ranging between 15.5 for social functioning (95% Confidence Interval (CI) 10.1, 20.7) to 32.6 for general health (95% CI 24.0, 41.1).

**Conclusions:**

We found that KTRs have better HRQOL than HD patients in physical and mental components of the SF-36 scale including physical functioning, role-physical, bodily pain, general health, vitality, social functioning, role-emotional, and mental health. Further longitudinal research comparing HRQOL among KTRs and the general population may identify key modifiable factors associated with lower HRQOL among KTRs that are amenable to intervention.

## Background

Chronic kidney disease (CKD) is a growing global public health problem in terms of mortality, disability, and financial costs [1, 2]. Globally, around 13% of the general population have some form of CKD [3]. In 2016, around 1.2 million deaths and 35 million disability-adjusted life years were attributed to CKD globally, which represents an increase in mortality and disability by 98 and 62% since 1990, respectively [4]. A significant proportion of patients with CKD progress to end stage renal disease [5, 6]. Currently, renal dialysis and kidney transplantation are the only two types of treatment available for patients with end stage renal disease [7]. Globally, the number of dialysis patients is projected to increase to 4.9 million by 2030 [8]. According to the Global Observatory on Donation and Transplantation, 100,097 kidney transplants were performed worldwide in 2019, which reflects an increase of 4.8% since 2018 [9, 10].

The literature suggests that kidney transplantation improves survival rates among patients on renal replacement therapy [11, 12]. However, kidney transplantation requires long-term immunosuppressive therapy, which is associated with significant side effects (such as recurrent infection, metabolic disorder, renal toxicity, fatigue, and poor self-perception of physical appearance) that may negatively affect patient’s health-related quality of life (HRQOL) [13–16]. HRQOL is a significant independent predictor of hospitalization and mortality in patients on renal replacement therapy [17, 18]. HRQOL remains an important health outcome measure in renal transplantation recipients and in dialysis patients, which help clinicians and patients make rational decisions about the optimal choice of treatment modality. The literature shows mixed findings regarding whether kidney transplantation is associated with clinically important improvement in HRQOL [19–21]. In addition, research findings indicate that HRQOL among kidney transplant recipients (KTRs) is influenced by diverse factors, including perceived health status, mental health, and socioeconomic factors [15, 22, 23]. Research examining HRQOL among KTRs and hemodialysis (HD) patients in the Middle East is sparse. The aim of this study was to compare the HRQOL in KTRs and HD patients in the West Bank, Palestine.

## Methods

### Design, setting and patient recruitment

A cross-sectional study was conducted among KTRs and HD patients from two kidney dialysis units in the West Bank (Palestine Medical Complex in Ramallah and An-Najah National University Hospital in Nablus) between May and August 2017. The total number of kidney dialysis patients in these two units (*n* = 451) represented about 37% of all kidney dialysis patients (*n* = 1216) in the West Bank in 2017 [24]. HD patients were eligible to participate in the study if they were 18 years old or over and were undergoing HD for at least three months. KTRs were eligible to participate in the study if they were 18 years old or over and had functioning kidney transplant for at least on year. All eligible patients were invited to participate in the study while they were attending to their scheduled HD sessions or their outpatient follow-up visits. All patients who agreed to participate in the study signed a written informed consent and completed a self-reported questionnaire, which collected data on sociodemographic variables and HRQOL. The study was ethically approved by An-Najah National University Institutional Review Board.

### Sociodemographic variables

The following variables were collected: age (18–29, 30–60, and > 60 years), gender (female, male), education level (elementary, primary, secondary, and > secondary education), and residential area (city, village, and refugee camp), and renal replacement therapy type (kidney transplantation or HD).

### Assessment of HRQOL

The Arabic version of the 36-Item Short Form Health Survey (SF-36) [25] was used to measure participants’ perceived HRQOL. The SF-36 self-report survey is a widely used instrument to assess perceived HRQOL, which has been validated in several languages including Arabic [26, 27]. The SF-36 has been used to study HRQOL in people with different health conditions including KTRs and HD patients [28, 29]. The SF-36 assesses different domains of HRQOL using eight subscales (physical functioning, role-physical, bodily pain, general health, vitality, social functioning, role-emotional, and mental health) and two component summary measures are derived from the eight subscales (physical component summary (PCS) and mental component summary (MCS)). The PCS represents a summary of physical functioning, role-physical, bodily pain, and general health, where the MCS represents a summary of vitality, social functioning, role-emotional and mental health subscales. The number of questions pertaining to each subscale range from two for bodily pain and social functioning to ten for physical functioning, and the number of responses to each question ranges from two options (yes, no) to six-point Likert scale (none, very mild, mild, moderate, severe, and very severe). Each response option is numerically coded and then converted into a score of 0 to 100. A mean score for each subscale and the two component summary measures is computed, with higher mean scores indicating better perceived HRQOL [25].

### Statistical analysis

Numbers with percentages were used to summarize sociodemographic variables. The chi-square test was used to examine if there are any statistically significant differences in sociodemographic variables between KTRs and HD patients. The renal replacement therapy type (Kidney transplantation or hemodialysis) and sociodemographic characteristics were modeled as predictors of raw mean scores of SF-36 subscales, PCS, and MCS. Multivariable linear regression was used to assess the relationships between sociodemographic variables and PCS and MCS scores among KTRs and HD patients separately. Similarly, multivariable linear regression was used to assess if there were any differences in HRQOL scores between KTRs and HD patients while adjusting for sociodemographic characteristics. The HRQOL scores for all subdomains, PCS and MCS were not normally distributed. Therefore, we used bootstrap sampling and estimation method, with 1000 repetitions, in all regression analyses. All inferential statistical tests were two-sided. A *P*-value less than 0.05 was considered statistically significant. All analyses were performed using IBM SPSS Statistics computer program (version 26.0, IBM Corp).

## Results

Three hundred and seventy-two patients participated in the study (100 KTRs (26.9%) and 272 HD patients). The sociodemographic characteristics differed significantly between the two groups for all variables except for residential area (Table 1). KTRs were younger than HD patients; 36 and 6.3% of KTRs and HD patients aged 18–29 years, respectively. About 75 and 58.1% of KTRs and HD patients were males, respectively. Higher proportion of KTRs (72%) obtained secondary school education or higher compared to HD patients (39.3%).

**Table 1:** Sociodemographic characteristics of kidney transplant recipients and hemodialysis patients

Variable	Kidney transplant recipients (n = 100), number (%)	Hemodialysis patients (n = 272), number (%)	***p-***value
**Age group (years)**			
18–29	36 (36.0)	17 (6.3)	< 0.001
30–60	61 (61.0)	151 (55.5)	
> 60	3 (3.0)	104 (38.2)	
**Gender**			
Female	25 (25.0)	114 (41.9)	0.002
Male	75 (75.0)	158 (58.1)	
**Education level**			
Elementary	23 (23.0)	52 (19.1)	< 0.001
Primary school	5 (5.0)	113 (41.5)	
Secondary school	40 (40.0)	48 (17.6)	
> Secondary	32 (32.0)	59 (21.7)	
**Residential area**			
City	33 (33.0)	76 (27.9)	0.166
Village	58 (58.0)	151 (55.5)	
Refugee camp	9 (9.0)	45 (16.5)	

### Sociodemographic factors associated with HRQOL among KTRs and HD patients

Studying detailed associations between sociodemographic variables and HRQOL scores at SF-36 subscales level was not the focus of the current study. Therefore, we report here only adjusted associations between sociodemographic variables and PCS and MCS scores (detailed data on this is available from the authors upon reasonable request). As shown in Table 2, HD patients aged more than 60 years had significantly lower PCS and MCS scores by 21.2 (95% confidence interval 9.2, 32.2) and 11.5 (95% CI 1.2, 22.0) than HD patients aged 18–29 years, respectively. No statistically significant difference in PCS and MCS scores were found between HD patients aged 18–29 years and those aged 30–60 years. As compared to female HD patients, male HD patients had higher PCS score by 8.8 points (95% CI 3.0, 14.2) but no statistically significant gender difference was found in MCS scores (5.7; 95% CI -0.3, 11.2). HD patients with elementary and primary school education levels had lower PCS scores by 21.4 (95% CI 14.4, 28.8) and 12.1 points (95% CI 3.3, 21.7) than those with education level higher than secondary school, respectively. However, no statistically significant difference in PCS scores was observed between HD patients with secondary school education level and those with education level higher than secondary school. Similar differences in MCS scores were observed among HD patients according to education level (Table 2). No statistically significant differences in PCS scores were found in relation to sociodemographic factors. However, KTRs aged 30–60 years had higher MCS scores by 7.3 (95% CI 1.8, 12.9) than KTRs aged 18–29 years. In addition, KTRs with primary school education level had lower MCS scores by 11.0 (95% CI 2.5, 18.7) than KTRs with education level higher than secondary school. No other statistically significant differences in MCS were observed in relation to other sociodemographic characteristics (Table 2).

**Table 2:** Sociodemographic factors associated with physical and mental component summary scores in kidney transplant recipients and hemodialysis patients

Predictor	Kidney transplant recipients (n = 100)		Hemodialysis patients (n = 272)	
	PCS	MCS	PCS	MCS
	β (BCa 95% CI)	β (BCa 95% CI)	β (BCa 95% CI)	β (BCa 95% CI)
**Age group (years)**				
18–29	Ref	Ref	Ref	
30–60	4.8 (−1.2, 11.6)	7.3 (1.8, 12.9)	−8.8 (− 19.3, 1.8)	−3.6 (− 13.9, 6.8)
> 60	− 13.8 (−45.4, 37.0)	−9.6 (−22.2, 1.7)	−21.2 (− 32.2, − 9.2)	− 11.5 (− 22.0, −1.2)
**Gender**				
Female	Ref	Ref	Ref	
Male	0.2 (− 7.2, 7.6)	0.1 (− 7.4, 8.4)	8.8 (3.0, 14.2)	5.7 (− 0.3, 11.2)
**Education level**				
> Secondary	Ref		Ref	Ref
Secondary school	−4.3 (− 12.9, 5.0)	−5.1 (− 12.3, 2.8)	−7.7 (− 18.2, 1.4)	− 7.0 (−16.6, 2.9)
Primary school	−10.3 (−22.4, 2.0)	−11.0 (− 18.7, − 2.5)	−12.1(− 21.7, − 3.3)	−11.6 (− 20.7, −1.9)
Elementary	−8.4 (− 34.6, 9.4)	− 11.4 (− 37.5, 9.0)	−21.4 (− 28.8, − 14.4)	− 18.0 (− 26.4, − 8.4)
**Residential area**				
City	Ref	Ref	Ref	
Village	−0.5 (− 8.2, 8.7)	−6.1 (− 23.3, 8.9)	6.4 (− 0.8, 13.5)	6.5 (−1.2, 13.8)
Refugee camp	− 6.7 (− 23.5, 9.2)	1.8 (− 5.2, 9.1)	−0.01 (− 9.9, 9.7)	1.4 (− 8.5, 11.2)

*PCS* Physical component summary, *MCS* Mental component summary, *β* Regression coefficient, *BCa* Bias corrected accelerated, *CI* Confidence interval, *Ref* Reference category

### Differences in perceived HRQOL scores between KTRs and HD patients

**Fig. 1:**
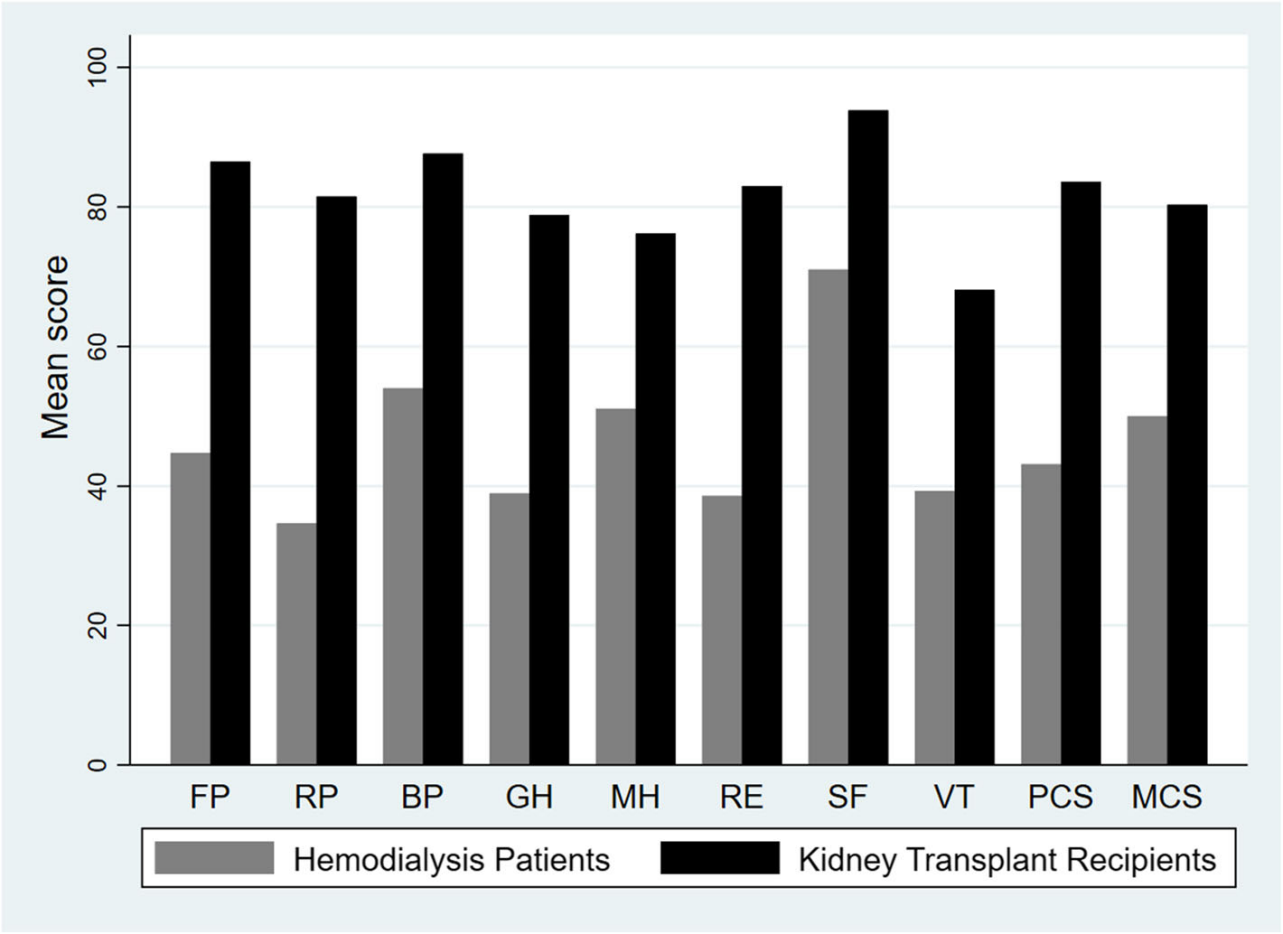
Unadjusted SF-36 mean scores in kidney transplant recipients (*n* = 100) and hemodialysis patients (*n* = 272). PF, Physical functioning; RP, Role-physical; BP, Bodily pain; GH, General health; VT, Vitality; SF, Social functioning; RE, Role-emotional; MH, Mental health; PCS, Physical component summary; MCS, Mental component summary

As shown in Fig. 1, KTRs had significantly higher HRQOL scores than HD patients in all SF-36 domains and subscales ranging from 22.8 (95% CI 17.1, 28.7) for social functioning to 46.8 (95% CI 38.3, 55.4) for role-physical.

After adjustment for sociodemographic variables (age group, gender, education level, and residential area), KTRs had significantly higher clinically important differences in all the SF-36 domains and subscales ranging between 15.5 for social functioning (95% CI 10.1, 20.7) and 32.6 for physical role (95% CI 24.0, 41.1) (Table 3).

**Table 3:** Adjusted differences in perceived HRQOL scores between kidney transplant recipients (*n* = 100) and hemodialysis patients (*n* = 272)

SF-36 dimensions	Group	β (BCa 95% CI)^**a**^	Mean score (95% CI)
Physical functioning	KTRs	24.5 (18.5, 30.2)	72.1 (66.7, 77.6)
	HDPs	Ref	47.7 (43.9, 51.4)
Role-physical	KTRs	32.6 (24.0, 41.1)	67.8 (59.8, 75.7)
	HDPs	Ref	35.2 (29.7, 40.6)
Bodily pain	KTRs	24.8 (18.2, 31.4)	79.6 (73.6, 85.5)
	HDPs	Ref	54.7 (50.7, 58.8)
General health	KTRs	32.4 (26.9, 38.3)	72.5 (67.2, 77.8)
	HDPs	Ref	40.1 (36.5, 43.7)
**Physical component summary**	KTRs	28.6 (23.6, 33.7)	73.0 (68.1, 77.8)
	HDPs	Ref	44.4 (41.1, 47.7)
Vitality	KTRs	20.5 (14.9, 26.6)	59.8 (54.6, 64.9)
	HDPs	Ref	39.3 (35.8, 42.9)
Social functioning	KTRs	15.5 (10.1, 20.7)	87.9 (82.2, 93.5)
	HDPs	Ref	72.4 (68.5, 76.3)
Role-emotional	KTRs	29.5 (20.2, 39.7)	68.6 (59.8, 77.3)
	HDPs	Ref	39.0 (33.0, 45.1)
Mental health	KTRs	24.5 (17.5, 30.6)	73.7 (67.5, 79.8)
	HDPs	Ref	49.2 (45.0, 53.4)
**Mental component summary**	KTRs	22.5 (17.6, 27.5)	72.5 (67.7, 77.3)
	HDPs	Ref	50.0 (46.7, 53.3)

*KTRs* Kidney transplant recipients, *HDPs* Hemodialysis patients, *SE* Standard error, *BCa* Bias corrected accelerated, *CI* Confidence interval, *Ref* Reference category. ^a^SF-36 HRQOL subscales and physical and mental component summary score estimates are adjusted for age group, gender, education level, and residential area

## Discussion

The present study compared HRQOL in KTRs and HD patients. As compared to HD patients, KTRs were more likely to be males, younger, and have higher education levels. These results are consistent with previous studies [30, 31]. Our study showed that KTRs have significantly better HRQOL than HD patients in all the SF-36 domains and subscales ranging from 15.5 for social functioning and 32.6 for physical role. These are considered clinically important differences in comparison to the minimal clinically important “benchmark” differences in HRQOL for any health condition (3–5 points) [32], stage five CKD (6–11 points) [33], and 15 points for patients with heart disease [34].

Our findings that KTRs have better HRQOL than HD patients in all domains of HRQOL agree with previous studies [35–39]. For example, Maglakelidze and colleagues reported that Georgian KTRs had significantly better HRQOL than HD patients in all SF-36 subscales ranging between 14.2 for social functioning and 33.6 for bodily pain [36]. These findings suggest the kidney transplantation is associated with significant improvements in HRQOL, which were also observed in previous cohort studies among KTRs [38, 39]. For instance, a two-year prospective cohort study from South Korea reported significant improvements in all SF-36 domains and subscales after kidney transplantation in comparison to baseline values [38].

The present study has several strengths. *First*, it included a relatively large number of KTRs and HD patients from two kidney units representing 37% of all patients on renal replacement therapy in the West Bank [24]. *Second*, the Palestinian population is highly homogeneous in terms of ethnicity, culture, spirituality, and physical environment. Therefore, the findings are likely to be generalizable to KTRs and HD in the West Bank or other areas in the Middle East with similar socioeconomic factors and healthcare systems. This study also has some limitations. One limitation is that self-reported perceived HRQOL is considered a subjective indicator of health status and may be influenced by individual expectations of health and recovery, which may underestimate or overestimate actual healthcare outcomes. A second limitation is that we collected no information on some factors associated with low HRQOL among patients with CKD, such as disease comorbidity, low body mass index, anemia, low glomerular filtration rate, and baseline HRQOL scores [37, 38]. However, we accounted for important sociodemographic variables associated with HRQOL among patients with CKD including age, gender, and education level. In addition, our findings are consistent with the findings of previous studies that accounted for those factors including socioeconomic, disease-specific factors, disease comorbidity and biochemical markers [36–38]. Another limitation is that our findings may not be generalizable to other populations and regions with different culture, religious beliefs, ethnicity, other socioeconomic factors, and healthcare systems influencing HRQOL. Finally, in the present study, we did not compare HRQOL between KTRs and a control group selected from the general population.

Further research comparing HRQOL between KTRs, patients treated with different renal replacement modalities, and the general population, with comprehensive assessment of factors affecting with HRQOL, may provide useful information about the magnitude of HRQOL attributed to kidney transplantation. This information may shed light on targeted interventions to address any modifiable factors associated with lower HRQOL among KTRs as compared to the general population.

## Conclusions

This study adds further evidence that KTRs have better HRQOL than HD patients in physical and mental components of the SF-36 including physical functioning, role-physical, bodily pain, general health, vitality, social functioning, role-emotional, and mental health. Further longitudinal research comparing HRQOL among KTRs and the general population may identify key modifiable factors associated with lower HRQOL among KTRs that are amenable to intervention.

## Data Availability

The datasets used and/or analysed during the current study are available from the corresponding author on reasonable request.

## Abbreviations

CI: Confidence interval
CKD: Chronic kidney disease
HD: Hemodialysis
HRQOL: Health related quality of life
KTRs: Kidney transplant recipients
MCS: Mental component summary
PCS: Physical component summary
SF-36: 36-Item Short Form Health Survey
